# The Use of Arsenic for the Destruction of the Dental Pulp

**Published:** 1900-04-15

**Authors:** O. L. Beard


### THE USE OF ARSENIC FOR THE DESTRUCTION OF THE DENTAL PULP.

BY DR. O. L. BEARD.

Read at a Meeting of the Cincinnati Odontological Society, January 26, 1900

When we have decided it has become necessary, for any cause, to remove the pulp from a living tooth, the great desideratum in the case is to accomplish this with the least amount of pain and by the quickest possible method.

Accepting this as being true, I believe the use of arsenic to be the most conservative method for the dentist and the most acceptable to the patient.

The employment of arsenic for the destruction of tooth pulps was originated by Dr. John R. Spooner, of Montreal, and we are indebted to his brother, Dr. S. Spooner, of New York, for its introduction to the profession in a treatise published by him in 1835 or 1836, and it might be truthfully said that the introduction of arsenic has done as much for the advancement of the dental profession to its present status as any other single event in our history.

The consensus of opinion seems to be that arsenic is a caustic irritant and escharotic, and that the death of the pulp is brought about by an engorgement of blood which produces a thrombus or plug at the apex of the root thus bringing about continued paralysis and death of the pulp.

Very much has been written and said concerning the use of arsenic in the teeth, and the subject has doubtless been well discussed from both sides.

It has been, and is to-day, I believe, maintained by many that the use of arsenic is a method attended by too many dangers and too much pain for the treatment of the dental pulp, and more especially do they consider it a method of the empiric and charlatan, since we have modern surgical means for its removal.

These modern surgical means are not within the province of this paper, but they refer, no doubt, to the different forms of anesthesia for the immediate removal of the pulp while the nerve itself has been made insensible by the use of cocain, or the patient is under the influence of some general anesthetic.

The opponents of the use of arsenic, aside from maintaining that the use of an anesthetic is the quickest and consequently the happiest solution of the exposed pulp difficulty, claim that the patient suffers untold agonies for the first few hours immediately following an application of arsenic to the nerves, and that if for no other reason than this its use should be discontinued.

From the earliest period of the use of arsenic for pulp devitalization there have been objections made to its employment on the allegation that a portion of the poison may attack the peridental membrane, and according to Dr. Louis Jack, all the disturbances which follow the removal of the pulp by this means have been attributed to the use of arsenic.

There does not, however, appear to be just cause for such accusation, especially if the action of the application has been rather closely observed and the pulp removed before the poison reaches beyond the pulp canal, and yet the time of retention seems to have no determined limits.

Many single-rooted teeth have been found to be insensible to pain after twenty-four hours have passed, and applications of arsenic have been retained in bicuspids and molars, through negligence of the patient, for many months without injurious effect. So it would seem that what injurious effect we find comes from the application of an unnecessarily large amount of arsenic, a part of which finds its way to the exterior tissue by capillary action after it has effected complete devitalization of the pulp.

Dr. Louis Jack tells us that when we have disturbances following the removal of the pulp by the use of arsenic they may be attributed to two causes: one being the effect of the arsenic, the other being due to the fact that the blood which supplied the pulp has, by the removal of this tissue, been distributed to the membrane surrounding the point of the root.

Dr. Wm. Donnelly, of Washington, in discussing a paper which was presented to the union meeting in Washington in June, 1899, by Dr. Wm. A. Mills, of Baltimore, said that the toxic effect of arsenic is very often shown in the peridental membrane, not immediately after its use but many years after, resulting in a difficulty that was hard indeed to combat, and Dr. Smith, of Baltimore, speaking at the same meeting and along the same line, said he had very often to regret the use of arsenic, as he had found alveolar conditions following its use which were not by any means to the credit of those making such application.

There are many different formula for nerve paste which include arsenic as their active ingredient for the destruction of the dental pulp, the most prominent being combinations of arsenic, morphia and carbolic acid or wood creosote.

Dr. E. C. Kirk gives the following formula which he states gives uniform satisfaction as a prompt obtunder and immediate relief from pain:

℞ Arsenious Acid;
Cocain Hydrochlorate, aa xx gr.;
Menthol Crystals, - - - v "
Glycerine, q. s. ad. to make a suitable paste.

Dr. James Truman recommends the following mixture which he says is prompt and painless: Take the amount of arsenic it is proposed to employ, add an equal amount of iodoform and make solution with 5 percent carbolic acid.

There are many other mixtures which do not differ materially from the ones mentioned.

I referred a moment ago to a paper read by Dr. Wm. A. Mills, of Baltimore, before the union meeting in Washington in June, 1899. He gave in this paper a formula which he has been using for some years in which he claims to control the irritating action of arsenic by the use of modifying drugs and to produce, without pain, the death of the pulp by its toxic effect:

℞ Arsenious Acid, - - - xx gr;
Morphine Acetatis, - - - v "
Powdered Alum, - - - x "
Beachwood Creosoti, - - gtt x.
Mix.

He claims the escharotic action of the arsenic is controlled by the use of creosote; the blood in the capillaries is driven out by the alum and creosote; the dental pulp is narcotized with creosote and morphia. The escharotic action of the arsenic being reduced it is slowly absorbed while the dental pulp is in a state of anesthesia and an unconscious death follows by poisoning, and not by irritation and congestion. He further says that when we have obtained the knowledge of how much arsenic to use and how long to leave the application in each case, we are then the masters of the situation.

Now, in opposition to all the evidence of the value of these combinations for the destruction of the dental pulp, I must say that I have, for the most part if not altogether, found them valueless, or, at least, unsatisfactory. I have not, however, tried the formula which Dr. Mills gives us.

For several years I used the different combinations of nerve paste in my effort to produce painless destruction of the dental pulp, but my patients almost invariably complained of severe pain and I have oftentimes had to remove the application after thirty or forty minutes' time, and I do not think this trouble due to excessive pressure, nor am I prepared to attribute it to the modifying drugs, as Dr. Mills calls them.

For four or five years my plan has been to invariably expose the nerve, if it is not already so—not a large exposure at the first sitting, but just enough to show that it has been punctured. I have been very unsuccessful in using arsenic in the teeth when the nerve was not exposed and the pulp punctured. To this exposed surface, then, I apply, by the use of a very small instrument, about the

1-100 part of a grain of powdered arsenic, without any modifying drugs, over which I place a small pledget of cotton saturated with carbolic acid, over this I place a small piece of dry cotton and seal the cavity with gutta-percha or cement, or sandarach and cotton, using, in most cases, the latter. While there may be some objection to using sandarach for this purpose, I have found it very satisfactory in my hands. The patient is instructed to return in forty-eight hours, at which time I open the pulp chamber well and with a smooth Donaldson broach I determine how far into the canal the arsenic has taken effect. If I can go well toward the end of the root without causing pain I apply a saturated solution of cocain, by the aid of which I find the nerve can be readily removed. If the first application of arsenic, however, does not destroy the sensation of pain well into the canal, I make a second application which is allowed to remain forty-eight hours, at the end of which time I very rarely find the nerve sensitive enough to interfere with its removal. It is a very rare thing that a patient has complained of any pain whatever after the first shock of the operation, which lasts but a few seconds. I find also that applications of arsenic made to an inflamed dental pulp will, instead of increasing the pain, in nearly all cases relieve the pain that already existed. If a tooth is aching severely I often make an application of very strong solution of cocain for a few moments until the severe pain passes away, after which I apply arsenic.

The scientific principles which are involved in the results I have obtained since using this method I am unable to give you, but I know that since using powdered arsenic alone the results have been most satisfactory indeed, and, in view of my experience, I am sure I can say with Dr. Mills, that I have found arsenic a tried and true friend in many puzzling circumstances, and that arsenic, like fire, is a good servant but a bad master.

DISCUSSION.

Dr. M. H. Fletcher: In applying arsenic to a pulp I try to differentiate between those which have still sufficiently active circulation and those which are in a gangrenous state. My experience is that we may have a condition of the pulp in which the circulation is almost stopped, and yet have abnormal sensitiveness. In other words, in the gangrenous pulp you may have a nerve filament remaining exceedingly sensitive, notwithstanding there is no circulation. In such cases we may apply arsenic with no fear of exciting periosteal inflammation. As regards the matter of destroying pulps indiscriminately, I am still old-fashioned enough to believe that a pulp, delicate in its organization though it be, is like any other organized tissue. If you can protect it with some sheltering agent which secures to it its normal condition, you can save it alive. It is difficult to cap a pulp without exerting undue pressure upon it. To thoroughly sterilize it is difficult. A few bacteria of the proper kind, and a little undue pressure, and death of the pulp results.

In applying arsenic to partly dead pulps, the danger of it penetrating where it is liable to work mischief is practically naught. I use arsenic as a sterilizing agent in those tortuous root-canals into which it is impossible, effectually, to introduce a broach and remove the contents. I have left the arsenic in sometimes, filling over it. In many cases I believe a tooth filled in that way will go a lifetime without giving trouble. A few crystals of arsenic will keep a tooth sterilized for many years.

Dr. Hunter: The definite action of arsenic I think is not known. Dr. J. Foster Flagg demonstrated by experiment, thirty-five years ago, that arsenic does not enter into the circulation of the pulp. He formulated a theory according to which the pulp is destroyed by strangulation. In the course of his experimenting he removed, I think, something like a hundred pulps which had been devitalized by arsenic, and tested them for the purpose of determining whether or not they showed the presence of the drug. He found a small portion in the parts immediately in contact with the drug, but none in the deeper tissue. I have never used arsenic, as arsenic, but have used the cobalt ore from which arsenic is derived or with which it is found in combination. I see no objection to leaving the arsenic in contact with the pulp indefinitely. I want the pulp to slough. Dr. Harlan and others have experimented to show the endosmotic penetrating, and others, tendency of arsenical application; but, so far as I know, it has not been even thus demonstrated that any considerable portion is absorbed. Dr. Flagg used a pellet of cotton saturated with arsenical solution, the pellet being no larger than a pinhead, to kill a dozen pulps. It was as effective in the last case as in the first.

Dr. H. T. Smith: Dr. Truman, in reviewing the progress of dental science, refers to the discovery of the use of arsenic for devitalizing dental pulps as one of the three most important events of the century. He says of this use of arsenic that it marks an epoch in the advancement of the profession, to so prominent a place does he assign the discovery. No other agent will take the place for devitalizing. Of course in certain cases the removal of the pulp without its use is practicable.

A recent author has called attention to the fact that the specific gravity of the arsenical preparations sold by the depots being greater than that of the other ingredients (of the pastes?), the arsenic settles to the bottom after a time, so that after about a year the mixture will be found to have lost its effectiveness.

Dr. Kumler: In my limited experience in the use of arsenic I have used with the paste a combination of antikamnia. It seems to me I have had less trouble with aching since I adopted the use of antikamnia. It does not interfere with the action of the arsenic.

Dr. W. S. Locke: I think it better to seal in an arsenical preparation with cement. If sandaric varnish be used there is a liability of leakage of the arsenic preparation before the patient returns for further treatment. Thus, injury to the adjacent soft tissues, through absorption of the arsenic, may be averted.

Dr. Rauh: Dr. Beard says that by securing an actual exposure of the pulp you can be sure of devitalizing the same painlessly. I find it impossible in many cases to so control the patient as to cut sufficiently to get a full exposure.

Dr. F. A. Hunter: What theory have you, Doctor Kumler, whereby you assume the efficacy of antikamnia in combination with arsenic, for killing pulps?

Dr. Kumler: It is a sedative.

Dr. Hunter: Used topically?

Dr. Kumler: Yes, sir; that is my theory. So far as my experience goes, it is more than theory. I use the two agents in equal proportions.

Dr. A. G. Rose: Five or six years ago, Dr. Fletcher filled three roots of teeth for me by this method, using arsenic combined with prepared chalk for the tortuous roots. They were pretty sore that night, so that I got up and smoked two or three cigars, and thought it over meanwhile. I thought several things of Dr. Fletcher. They got all over the soreness in less than a day and have remained comfortable ever since.

An effective combination for destroying pulps is one part arsenic and two or three parts acetate of lead. The latter agent acts as a sedative. Very little pain results. A remedy for neutralizing arsenic accidentally escaping upon a soft tissue is dyalized iron.

Dr. Stern: Arsenic has no escharotic effect unless in contact with moisture. Dyalized iron may be applied around the edges of a sealed cavity containing arsenic, as a precaution against poisoning the surrounding tissue. In combination with arsenic it yields arsenate of iron.

Dr. F. W. Sage: I am not at all sure that the dyalized iron, used in that way, would so combine with the arsenic, presuming any had escaped. I seriously doubt it, indeed. I made some investigation of the matter several years ago, and, while I do not now recall the definite results of my inquiry, I know that the opinion I got was that this preparation of iron is of doubtful efficacy for the purpose mentioned.

Before applying arsenic, dry the cavity thoroughly, and thus avoid the caustic effect of the drug and consequent pain.

Dr. J. R. Callahan: I am much pleased with the paper and have received a good deal of information from the discussion. I fear that the use of this agent is not always accompanied with proper care. I adjust the dam and make a preliminary filling, to so enclose the arsenical application as to form a pocket, so precluding danger of any escaping beyond the bounds of the cavity. I think the various combinations for the prevention of pain unnecessary. Use carbolic acid or oil of cloves to make a paste. Let the application remain a week or more. Keep it sealed securely and without pressure in the tooth, and your patient will have no pain.

Dr. H. A. Smith: I am reminded by the Chairman, Dr. Heise, of a remarkable statement made at the last Niagara Falls meeting. It refers to the sealing in of devitalizing applications, or other applications, in teeth. Cotton and sandarach is a "sloppy" way of sealing, condemned on high authority. Dr. Black stated at the meeting in question that oxyphosphate fillings shrink sufficiently to allow of arsenical applications escaping.

This, if it be true, is a revelation to many of us, and condemns that material equally with cotton and varnish for this particular purpose. Gutta-percha is a better sealer. An interesting question is: how long after arsenic has been brought into contact with tissue does it continue to act? I do not think its action is unlimited as to time. It must be satisfied in a short time. Is its effect soon exhausted? In the so-called arsenical ulcer, does the arsenic continue to act after the soft tissues have sloughed away and ostitis has set in? Evidently it does not act by strangulation, for obviously there can be no strangulation in those cases where a tooth, undeveloped, having a large, open foramen, still has its pulp destroyed if you apply the agent.

Undoubtedly one of the most notable discoveries of the century in dentistry is Dr. Spooner's discovery of the applicability of arsenic for devitalizing tooth-pulps. We do not perhaps quite appreciate how important a discovery it is. A high authority says of it: "The good accomplished by this agent is unparalleled."

As to the various compounds of arsenic, it is the manner of the use of the agent which is important. Cocain often prevents any pain when used in the combination. I think it has no control over the vaso-motor nerves, but exerts its influence upon the sensory nerves particularly. Its secondary effects would be on the vaso-motor nerves.

Dr. Fletcher: The presence of arsenic in minute roots prevents micro-organisms from forming at all. The moment they seek to invade the root through the foramen there is the particle of arsenic to meet and combat them. Chalk is used in my operations merely as a vehicle or diluent.

Dr. Stern: Dr. Hunter's use of cobalt is only another use of arsenic. It is less painful than arsenic, probably because comparatively it contains but little arsenic. Ordinarily one-thirtieth of a grain of arsenic is enough to devitalize a pulp.

Dr. Callahan: I think it was Dr. Atkinson who called my attention to the use of a coloring powder with arsenic to enable him easily to distinguish the arsenic when using it in a tooth.

Dr. O. N. Heise: Dr. W. D. Miller published in *The Cosmos*, in 1894, I think it was, the results of experiments he made on mice, inoculating with arsenic the ends of the tails, which had first been encased in glass rings and plaster of paris. He found that the arsenic caused œdema and the usual symptoms of inflammation which caustic action produces, up to the rings; beyond, there was no action of the drug observed. He pronounced the action decidedly caustic. That, he claims, is the occasion of its efficacy. Arkovy also has made complete experiments in this same line.

Dr. Beard: I will say, in reply to Dr. Rauh's inquiry, that I do not always succeed in exposing the pulp before applying arsenic, as I would like to do. In many cases, however, I succeed by the use of cocain. I have had little success in devitalizing unexposed pulps, pulps covered with a layer of dentin.

Dr. Hunter: I had supposed that it was generally conceded that a pulp covered with a layer of dentin could nevertheless be devitalized. It is only a question of leaving the application long enough.

Dr. Heise: Ferro-pyrine is a good remedy for neutralizing arsenic.

Binz suggests that arsenic is effective as a protoplasm poison, because of its facility for setting free nascent oxygen, which is the active agent. He calls it "oscillation," because the arsenious acid, when applied to living structures with heat, moisture and free access of air, is changed by oxidation to arsenic acid ($AS_2O_5$), and then back to arsenious acid ($AS_2O_3$).

Arkovy, after a series of careful investigations, concludes that arsenious acid, applied to a tooth pulp, induces inflammatory hyperæmia, expanding blood-vessels and tendency to thrombosis, no coagulation, apparent chemical reaction with the hæmoglobin in molecular form; the connective tissue cells are irritated and swollen; the odontoblast cells are unaffected, but great degeneration of the axis-cylinder of the nerve fibers.

Miller (*Cosmos*, page 676, 1894) found, by a series of experiments on mice, that although great œdema followed the applications of arsenic, there was no conclusive evidence that pulps were devitalized by strangulation, and there was no destructive cauterant or escharotic action. He made combination of various cauterant and antiseptic drugs with arsenious acid, and from his experiments concludes that the rapidity and intensity of arsenical applications depend on the conditions and the drugs combined with the arsenic. In restricted exposures of the pulp astringent cauterants are contra-indicated. In obstinate cases he recommends that either oil of cloves or glycerine or salt solutions of arsenious acid be used, undiluted by any third constituent. He recommends thymol in place of morphine as the sedative. And to prevent dissemination in deciduous teeth, or to sterilize and embalm portions of pulp which must remain in the teeth, use in connection with the arsenious acid either thymol, oxide of zinc, or iodoform.

We have found in our own experience that nerve pastes containing carbolic acid produce a soluble residuum, while those containing chloride of zinc and oil of cloves, or Formalin preparations, produce a dry or hard residuum.—[EDITOR.

